# Hyperparathyroidism presenting as hyperemesis and acute pancreatitis in pregnancy: A case report

**DOI:** 10.1097/MD.0000000000025451

**Published:** 2021-04-09

**Authors:** Wen-Hsuan Tsai, Chun-Chuan Lee, Shih-Ping Cheng, Yi-Hong Zeng

**Affiliations:** a Division of Endocrinology and Metabolism, Department of Internal Medicine MacKay Memorial Hospital, Taipei; b Department of Medicine, MacKay Medical College, New Taipei City; c Department of Surgery, MacKay Memorial Hospital, Taipei, Taiwan ROC.

**Keywords:** hyperemesis, hyperparathyroidism, pancreatitis, pregnancy

## Abstract

**Rationale:**

Nausea and vomiting are common in the early period of pregnancy and rarely seen as an overture to pancreatitis.

**Patient concerns:**

Here, we describe a 31-year-old pregnant woman who presented with progressive nausea and vomiting followed by severe epigastric pain. Biochemical data and sonographic features confirmed the occurrence of acute pancreatitis. Accompanying electrolyte abnormalities included hypercalcemia and hypokalemia. Her condition stabilized following medical treatment, but hypercalcemia persisted despite intravenous fluids and furosemide administration.

**Diagnoses:**

A diagnosis of primary hyperparathyroidism was made based on the elevated parathyroid hormone level and urinary calcium-to-creatinine clearance ratio.

**Interventions:**

Localization study with neck ultrasonography indicated left inferior parathyroid adenoma. She underwent parathyroidectomy successfully and made an uneventful recovery.

**Outcomes:**

At 37 weeks of gestation, she had a serum calcium level of 8.8 mg/dL and normal parathyroid hormone of 28.55 pg/mL. A healthy baby weighing 3180 g was delivered smoothly with no clinical nor biochemical evidence of hypocalcemia.

**Lessons:**

Although primary hyperparathyroidism during pregnancy is usually asymptomatic, patients may present with atypical manifestations such as hyperemesis and pancreatitis. Proper diagnosis and timely intervention are crucial to minimizing potential hazards to both mother and fetus.

## 1 Introduction

Nausea and vomiting are very common in the early period of pregnancy. While most mild to moderate sickness resolves after the first trimester, the severe form, or hyperemesis gravidarum, is less common and might persist throughout pregnancy.^[1]^ Hyperemesis gravidarum may lead to acid-base imbalance and increase the need for hospitalization.^[2]^ When additional gastrointestinal symptoms are present, other causes of nausea and vomiting should be considered.

Acute pancreatitis is a rare condition in pregnancy, but it may result in catastrophic maternal and perinatal morbidity and mortality. Causes of pancreatitis included gallstones, alcohol abuse, hypertriglyceridemia, and medication. In general, pancreatitis is an uncommon manifestation of hyperparathyroidism. Pancreatitis induced by hyperparathyroidism-associated hypercalcemia during pregnancy is even further rare. Very few cases have been anecdotally reported in the literature.^[3–5]^

Here, we present a case of hyperparathyroidism presenting as hyperemesis and pancreatitis during pregnancy. The patient was initially managed conservatively. Nonetheless, parathyroidectomy turned out to be necessary, and the operation was undertaken successfully without any maternal or fetal complications.

## 2 Case presentation

Institutional review board approval (20MMHIS057e) for this report was obtained from MacKay Memorial Hospital. Informed consent was obtained from the patient for the purpose of publication. All data generated or analyzed during this study are included in this published article.

A 31-year-old woman, gravida 2 para 1, presented at 15 weeks’ gestation with severe epigastric pain. She had progressive nausea, vomiting, polyuria, and general malaise for one week prior to admission. Past medical history was significant for three episodes of urolithiasis, which was treated by extracorporeal shock-wave lithotripsy. She had a prior cesarean delivery at 37 weeks because of prolonged labor. Otherwise, the patient's past medical and family history was unremarkable. She drank alcohol occasionally and did not consume alcoholic beverages during this pregnancy.

On initial examination, the patient was asthenic but oriented to person, place, and time. Her supine blood pressure was 103/66 mmHg, pulse was 83 beats/min, body temperature was 37.1°C, and respiratory rate was 20/min. Her head and neck examination was unremarkable. The bowel sounds were hypoactive, and diffuse abdominal tenderness was present without rebound, guarding, or ascites. Pelvic and ultrasound examinations corresponded to 15 weeks of pregnancy. No uterine contractions were noted, and the fetal heart rate appeared normal.

The patient's initial laboratory profile is presented in Table 1. Biochemical investigations showed remarkable electrolyte abnormalities, including hypokalemia, hypomagnesemia, hypercalcemia, and hypophosphatemia. Significantly elevated amylase and lipase were noted. Electrocardiography showed sinus rhythm and normal PR and QT intervals. A diagnosis of acute pancreatitis was made based on the clinical presentation and the laboratory findings.

**Table 1:** Laboratory profile at the initial presentation.

Parameter	Patient's value	Reference range
Hemoglobin	9.6	11–16 (g/dL)
Total white blood cell	10700	4000–10000 (/μL)
Platelet	290	140–450 (×10^3^/μL)
Glucose	113	70–99 (mg/dL)
Albumin	3.3	3.5–5.0 (g/dL)
Total bilirubin	1.0	0.3–1.2 (mg/dL)
Aspartate aminotransferase	26	15–41 (IU/L)
Alanine aminotransferase	11	14–40 (IU/L)
Alkaline phosphatase	301	38–126 (IU/L)
Triglyceride	172	35–150 (mg/dL)
Blood urea nitrogen	6	8–20 (mg/dL)
Creatinine	0.6	0.4–1.2 (mg/dL)
Sodium	132	135–144 (mEq/L)
Potassium	2.2	3.5–5.1 (mEq/L)
Chloride	103	101–111 (mEq/L)
Total calcium	12.9	8.9–10.3 (mg/L)
Ionized calcium	2.17	1.20–1.38 (mmol/L)
Phosphorus	1.1	2.7–4.5 (mg/L)
Magnesium	0.8	1.8–2.5 (mg/L)
Amylase	1615	28–100 (U/L)
Lipase	1609	22–51 (U/L)
pH	7.450	7.350–7.450
Bicarbonate	26	20–26 (mmol/L)
Intact parathyroid hormone	490.2	10.0–60.0 (pg/mL)
Thyroid-stimulating hormone	0.81	0.25–4.00 (IU/mL)
25(OH)D	5.55	30–100 (ng/mL)

She was managed with fasting, intravenous fluids, electrolyte repletion, analgesics, and anti-emetics. To investigate the etiology of pancreatitis, abdominal ultrasonography showed an edematous and enlarged pancreas, without signs of gallstones nor cholecystitis. Common bile duct dilatation or adrenal tumor were not seen.

**Figure 1:**
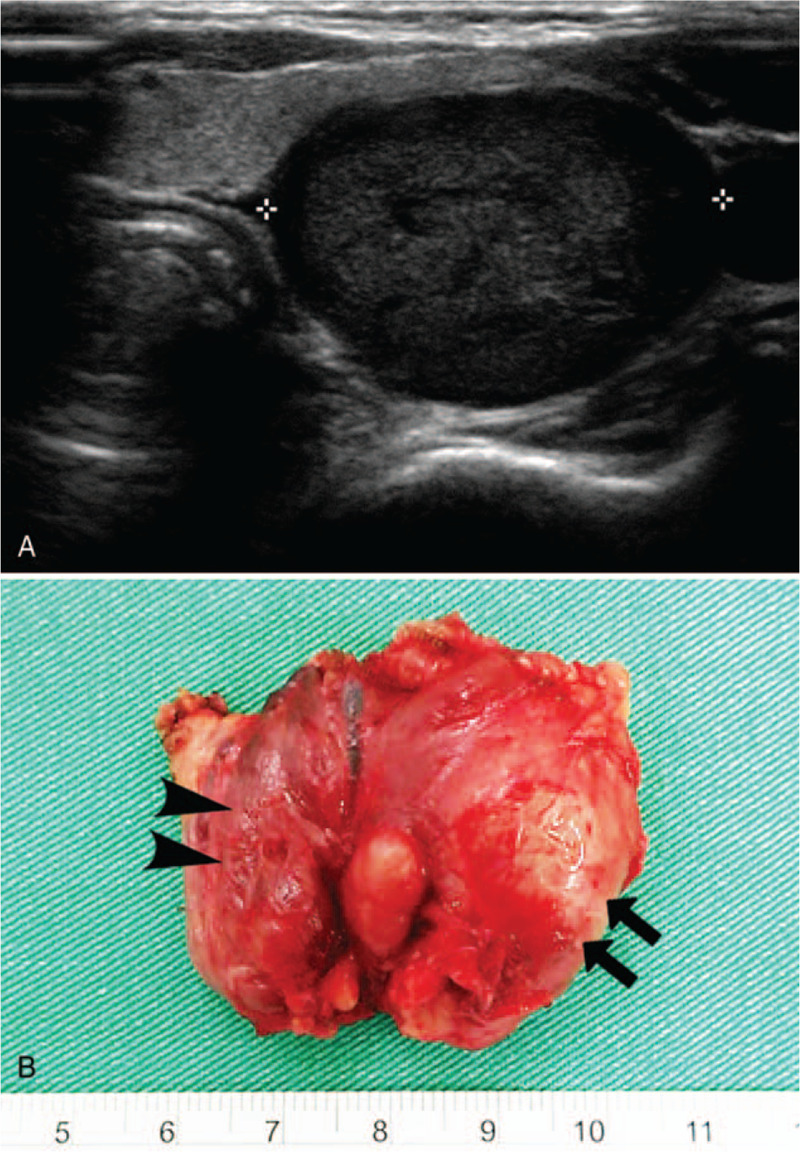
Clinical features of the parathyroid tumor in our patient. (A) Neck ultrasonography revealing a hypoechoic mass adjacent to the left thyroid lobe; (B) Gross image of the resected specimen showing a parathyroid tumor (arrows) densely adhered to the left thyroid (arrowheads).

The patient's abdominal pain subsided and serum potassium levels normalized following therapy. However, her high serum calcium level persisted above 12 mg/dL. An intact parathyroid hormone (PTH) level was found to be elevated at 490 pg/mL with a low 25-hydroxyvitamin D level of 5.5 ng/mL. The urinary calcium-to-creatinine clearance ratio was 0.07. With the presumptive diagnosis of primary hyperparathyroidism, aggressive saline infusion and furosemide were administered. A neck ultrasound revealed a well-defined hypoechoic mass, measuring 29 mm × 23 mm × 15 mm, located posteriorly to the left thyroid lobe (Fig. 1A).

**Figure 2:**
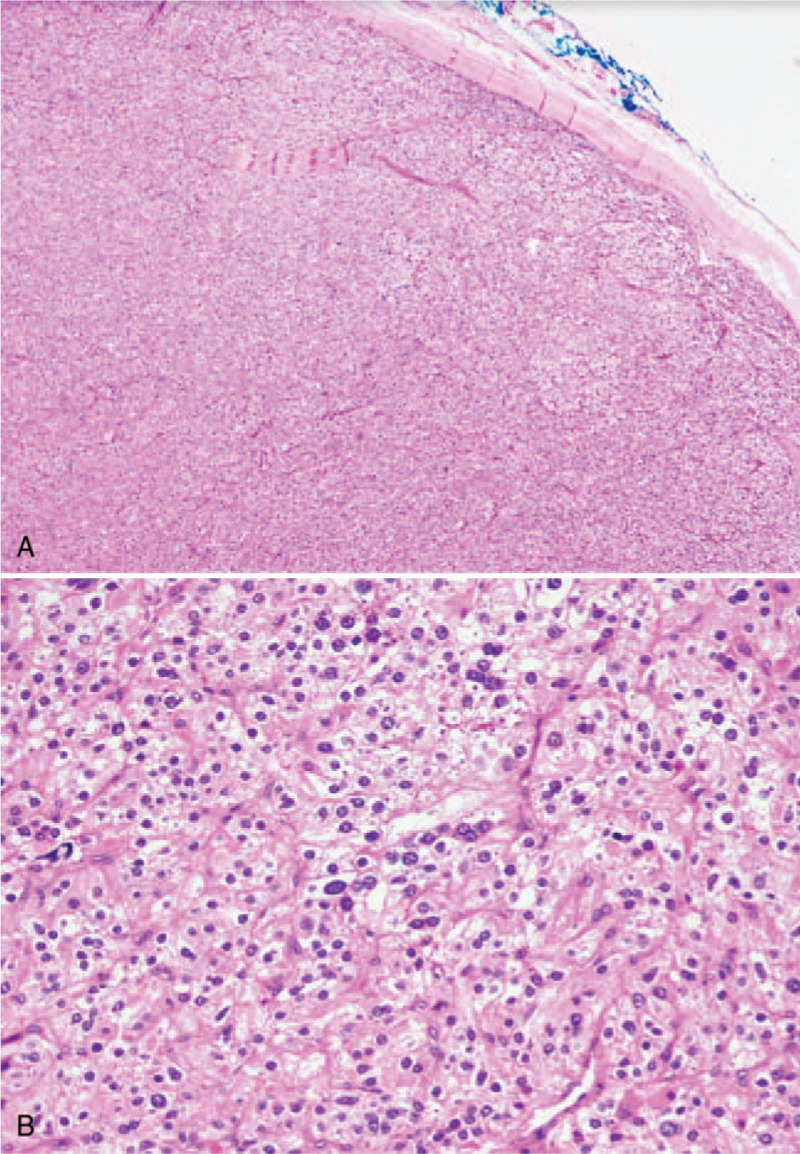
Pathologic features of the parathyroid tumor in our patient. (A) Low-magnification micrograph of the parathyroid mass composed of clear cells; (B) High-magnification view showing no increase of mitosis nor severe cellular atypia.

Despite aggressive medical treatment, her calcium levels remained poorly controlled at 13.1 mg/dL. A multidisciplinary decision was made to proceed with surgery. Seven days after admission, the patient was brought to the operating room, where she was intubated and placed under general anesthesia. An enlarged left inferior adenomatous parathyroid tumor was identified with dense adhesions to the thyroid. Concerning the possibility of parathyroid carcinoma, the parathyroid tumor and the left thyroid lobe were en bloc resected (Fig. 1B). Central neck dissection was not performed because lymph nodes were grossly normal. The remaining parathyroid glands were unremarkable. Histological examination was consistent with the diagnosis of parathyroid adenoma. The adjacent thyroid showed lymphocytic thyroiditis (Fig. 2A and B). Her calcium and PTH rapidly normalized after surgery. The calcium level on the next day dropped to 8.4 mg/dL, suggesting hungry bone syndrome. Intravenous calcium gluconate, as well as oral calcium carbonate and calcitriol, were started. The patient was discharged on the fifth postoperative day with her serum calcium maintained at 8.1 mg/dL and PTH at 7.5 pg/mL. The rest of the pregnancy was uneventful. At 37 weeks of gestation, she had a serum calcium level of 8.8 mg/dL and normal PTH of 28.55 pg/mL. A healthy baby weighing 3180 g was delivered smoothly with no clinical nor biochemical evidence of hypocalcemia.

## 3 Discussion

Primary hyperparathyroidism predominantly affects women, typically in the first decade after the menopause.^[6]^ It is infrequently seen in women of childbearing age and therefore tends to be neglected in the differential diagnosis, particularly for those patients presenting with atypical symptoms. The incidence of primary hyperparathyroidism in women of childbearing age has been estimated to be 6 to 8 cases per 100,000 population per year.^[7]^

Symptoms of hyperparathyroidism during pregnancy are usually mild or easily misdiagnosed as pregnancy-related sickness. An Israeli study suggested that pregnant women with primary hyperparathyroidism had only mildly elevated serum calcium levels, and there was no increased risk of obstetrical complications.^[8]^ A register-based cohort study from Denmark also demonstrated that a diagnosis of primary hyperparathyroidism did not increase the rate of abortions.^[9]^ A plausible explanation is that parathyroid tumors had the expression of functional estrogen receptor (ER)-β but not ER-α.^[10]^ Furthermore, expansion of the intravascular volume and fall in serum albumin levels may mask hypercalcemia in pregnancy.^[11]^

On the contrary, other studies have shown that hyperparathyroidism during pregnancy may be associated with a higher risk of preeclampsia and fetal loss.^[12,13]^ In extreme cases of hypercalcemic crisis, altered mental status or seizure may develop. Neonates born to mothers with untreated hyperparathyroidism might have neonatal hypocalcemia and tetany owing to parathyroid suppression in utero.^[14]^ In early reports, transient hypocalcemia shortly after birth was frequently observed in affected infants.^[4]^

Hypercalcemia induced by hyperparathyroidism contributes to the disruption of intracellular calcium homeostasis and premature trypsinogen activation of pancreatic acinar cells.^[15]^ Pancreatitis is virtually not seen in patients with mild hypercalcemia. Considering that most pregnant patients with hyperparathyroidism have subtle manifestations, hyperparathyroidism-associated pancreatitis is extremely rare.^[3–5]^ Pancreatitis in pregnancy is associated with maternal preeclampsia, preterm delivery, and intrauterine fetal death.^[16]^ It is noteworthy that non-gallstone pancreatitis may result in more complications and poor outcomes. Maternal death related to pancreatitis has been sporadically reported.^[3,17]^

Other than abdominal pain, nausea and vomiting are frequently reported symptoms in hyperparathyroidism-associated pancreatitis during pregnancy.^[5]^ Although nausea and vomiting are common in early pregnancy, accompanying abdominal pain is exceptionally rare. Differential diagnoses include gastroenteritis, appendicitis, cholecystitis, peptic ulcer, intestinal obstruction, urinary tract infection, and gestational trophoblastic disease. For pregnant patients with hyperemesis, additional investigation is recommended when abdominal pain or signs of infection are present, or when symptoms begin after the first trimester.

Our patient also had marked electrolyte abnormalities. Hypokalemia in association with hyperparathyroidism during pregnancy has been reported and was found to be aldosterone-independent.^[18]^ Although the concentration of potassium in gastric secretions is low, vomiting can cause fluid loss and metabolic alkalosis, which are in turn accompanied by increased renal potassium excretion. About 40% to 70% of filtered magnesium is absorbed in the thick ascending limb of the loop of Henle. The process is regulated by the calcium-sensing receptor (CaSR). During hypercalcemia, activation of the basolateral CaSR results in calcium excretion and magnesium wasting.^[19]^ Magnesium deficiency will further exacerbate potassium wasting. In this context, the concurrent correction of multiple electrolyte deficiencies (hypomagnesemia and hypokalemia) is of utmost importance in these patients.

Parathyroidectomy is generally recommended for younger patients, even with mild primary hyperparathyroidism.^[20]^ Compared with surveillance, surgery may be associated with a better quality of life.^[21]^ Nonetheless, when the diagnosis of hyperparathyroidism is made during pregnancy, a careful follow-up without any medical treatment is appropriate for asymptomatic patients with mild hypercalcemia.^[7]^ Parathyroidectomy after the first trimester is recommended when the patient is symptomatic or when the serum calcium is higher than 12 mg/dL.^[22]^ An Australian study found that the rates of preeclampsia and preterm delivery were higher in medically treated patients than patients undergoing surgery.^[23]^ Bisphosphonates are contraindicated during pregnancy. Treatment with calcimimetics during pregnancy was reported,^[24,25]^ but the safety of cinacalcet in pregnant patients has not been established.

In conclusion, although primary hyperparathyroidism is uncommon in pregnancy, its presentation may be quite variable. In this patient, the associated hypercalcemia was promptly identified as the culprit behind her clinical hyperemesis and pancreatitis. Potential risks to both mother and fetus could be minimized with proper diagnosis and timely intervention.

## Author contributions

**Conceptualization:** Wen-Hsuan Tsai, Yi-Hong Zeng.

**Investigation:** Wen-Hsuan Tsai, Shih-Ping Cheng, Yi-Hong Zeng.

**Supervision:** Chun-Chuan Lee.

**Validation:** Wen-Hsuan Tsai, Shih-Ping Cheng, Yi-Hong Zeng.

**Writing – original draft preparation:** Wen-Hsuan Tsai, Shih-Ping Cheng.
